# Self reported experience of physical abuse in schools in Nigeria

**DOI:** 10.1192/j.eurpsy.2021.1888

**Published:** 2021-08-13

**Authors:** A. Oladosu, O. Abiodun, M. Tunde-Ayinmode

**Affiliations:** 1 Johnson Community Hospital, Lincolnshire Partnership Foundation Trust, Lincolnshire, United Kingdom; 2 Behavioural Sciences, University of Ilorin Teaching Hospital, Ilorin, Nigeria

**Keywords:** Child, physical abuse, school

## Abstract

**Introduction:**

Physical abuse of children in schools is common in Nigeria, but its prevalence and forms are yet to be clearly studied.

**Objectives:**

To determine the prevalence and pattern of physical abuse of children in Secondary Schools in Ilorin Nigeria

**Methods:**

Cross sectional survey of secondary school students aged 11-18 years in Ilorin Nigeria using multistage random sampling technique with proportional allocation was done. Respondents completed the ICAST-CI questionnaire which covers child abuse in educational institutions. Prevalence and pattern of child abuse was computed.

**Results:**

Table 1: Pattern of physical abuse in School in the last 12 months

**Table:** 

Form of abuse	Frequency	Percentage
Physical Abuse* (n=1,554)		
Caused you pain	1514	97.4
Stay in cold/heat	1285	82.7
Hit you with fist	716	46.1
Kneel in a way that hurts	686	44.1
Slap on head as punishment	663	42.7
Twist ear as punishment	635	40.9
Kicked you	476	30.6
Slap on your arm	448	28.8
Throws object at you	347	22.3
Crushed your finger as punishment	231	14.9
Choked you	224	14.4
Pulled your hair as punishment	116	7.5
Starvation as punishment	67	4.3
Forced to do dangerous acts	64	4.1
Soap or pepper in mouth	48	3.1
Cut you with sharp object	10	1.0
Burnt as punishment	-	-
Put into hot or cold water	-	-
Tied with rope or belt	-	-

**Conclusions:**

Physical abuse of children is extremly commonplace in public secondary schools in Nigeria. it is neccessary to explore other ways of dealing with children in educational institutions.

**Disclosure:**

No significant relationships.

